# LONELINESS AND COGNITIVE FUNCTION IN OLDER ADULTS: A LONGITUDINAL ANALYSIS

**DOI:** 10.1093/geroni/igae098.2889

**Published:** 2024-12-31

**Authors:** Elnaz Abaei, Peter Martin

**Affiliations:** Iowa State University, Ames, Iowa, United States; Iowa State University, Ames, Iowa, United States

## Abstract

Only a few studies have confirmed cognition as a predictor of loneliness. This study investigated the dynamic relationship between loneliness and cognitive performance in older adults through a random intercept cross-lagged panel model. Data from 8,443 individuals aged 65 years and older, drawn from Waves 9–14 of the Health and Retirement Study (HRS), were analyzed. Loneliness was assessed using the UCLA Loneliness Scale, and cognitive function was measured using delayed memory recall. Age, gender, and education were included as covariates. We combined waves 9-10, waves 11-12, and waves 13-14 to include all participants. Bivariate correlations revealed that older age was negatively correlated with cognitive function, loneliness was positively correlated with older age, and male gender and higher education were positively correlated with cognitive function, and negatively correlated with loneliness. Cognition and loneliness were negatively associated. Using Mplus, we computed an unconditional random intercept cross-lagged panel model (RI-CLPM) with no exogenous predictors (X2=.186, df=1, p=0.67, RMSEA=0.000 [.000,.018], CFI=1.0, TLI=1.0) and a conditional model (X2=13.431, df=7, p<.06, RMSEA=0.009 [.000,.015], CFI=.999, TLI=.997) including age, gender, and education as predictors. Results indicated individuals reporting higher loneliness exhibited lower cognitive functioning scores and older age. Male gender and higher education were negatively associated with loneliness and positively with cognitive function. This study underscores the link between loneliness and cognitive decline in older adults, emphasizing the need to address loneliness to potentially reduce cognitive decline. Insights into demographic predictors of loneliness and cognitive function could inform targeted interventions for promoting successful aging.

